# A Case of Common Iliac Artery Pseudoaneurysm Formation, Rupture, and Rectal Fistula Caused by *Escherichia coli*

**DOI:** 10.1002/ccr3.72078

**Published:** 2026-03-04

**Authors:** Xiaojie Zhao, Ling Zhang, Aiqin Liu

**Affiliations:** ^1^ Emergency Center of Lanzhou University Second Hospital Lanzhou China; ^2^ Department of Intensive Care Medicine Second Hospital of Lanzhou University Lanzhou Gansu China

**Keywords:** common iliac artery, Escherichia coli, pseudoaneurysm, rectal fistula

## Abstract

When septic shock is combined with acute lower gastrointestinal bleeding, the rupture and bleeding of a common iliac artery pseudoaneurysm caused by *Escherichia coli* infection should not be missed. Active surgery combined with strict anti‐infection strategies can save lives.

## 1 Introduction

Pseudoaneurysm is a rare vascular abnormality that is a cystic pulsatile hematoma formed by the leakage of blood outside the blood vessel and wrapped by the local surrounding fibrous tissue. It is mostly caused by trauma, infection, or iatrogenic injury. Different from a true aneurysm, the arterial wall of a pseudoaneurysm is partially ruptured, most often involving the intima and media, resulting in a hematoma around the artery [1]. Unruptured pseudoaneurysms may present as pulsatile masses but are usually asymptomatic. Rupture often manifests as abdominal pain, gastrointestinal bleeding, and other symptoms. In Western countries, mycotic aortic aneurysm (MAA) accounts for approximately 0.65% to 1.3% of all aortic aneurysms [2], whereas a higher incidence has been reported in East Asia. MAA typically presents as a pseudoaneurysm characterized by rapid expansion and a high propensity for rupture. Earlier reports have associated infectious aneurysms with pathogens such as *Treponema pallidum* (syphilis) [3], *Mycobacterium tuberculosis* [4], and Gram‐positive bacteria, including *Staphylococcus*, *Enterococcus*, and *Streptococcus pneumoniae* . Among Gram‐negative bacteria, Salmonella is more frequently implicated [5], while *Escherichia coli* ( *E. coli* ) remains a relatively uncommon cause [6], accounting for only about 3.1% of cases [7]. MAA due to *E. coli* most commonly arises from hematogenous spread secondary to urinary tract infections [8], though it may also result from septic embolization or direct extension from adjacent infectious foci, leading to arterial wall degradation and subsequent pseudoaneurysm formation [7].

Osteomyelitis refers to an infectious disease of the bones, mainly caused by the spread of bacteria through the blood, trauma, or infection from adjacent tissues. Lesions can be confined to bone tissue, commonly found in the vertebrae, long bones, pelvis, and sternum. It can also involve multiple sites such as the bone marrow, cortical bone, periosteum, and even the surrounding soft tissues. It is more common in *Staphylococcus aureus* infections, while *E. coli* infections are rare [9].

## 2 Case History and Examination

A 60‐year‐old male was admitted to the hospital with a one‐month history of intermittent melena and an acute episode of hematochezia for 12 h. Approximately one month before admission, he began experiencing intermittent passage of black stools, around 50 mL every 4–5 days, accompanied by lower back pain. Seven days before admission, he was diagnosed with lumbar intervertebral disc herniation at a local traditional Chinese medicine clinic and received acupuncture and moxibustion treatment. Twelve hours before admission, he developed sudden bright red rectal bleeding with blood clots, totaling approximately 500 mL, without hematemesis or fever. At that time, his blood pressure dropped to 80/40 mmHg, and he received active fluid resuscitation (details unknown; no blood transfusion administered). He was urgently transferred to our emergency department via ambulance.

Upon arrival, the patient was conscious but hypotensive (90/60 mmHg), with chills and shivering; body temperature was not recorded. Initial laboratory findings included: WBC 7.9 × 10^9^/L, neutrophils 88%, hemoglobin 87 g/L, RBC 3.08 × 10^12^/L, platelets 244 × 10^9^/L, and elevated CRP. Coagulation studies showed PT 16.8 s, PT% 49%, APTT 31.8 s, fibrinogen 3.62 g/L, and D‐dimer 6.81 μg/mL. An abdominal scan revealed sacral bone destruction and an anterior sacral mass, possibly indicative of a space‐occupying lesion or hematoma. The patient was treated with shock correction, hemostasis, and supportive care and was admitted to the general surgery ICU.

Past medical history was unremarkable for hypertension, diabetes, chronic diseases, or a family history of aneurysms. On admission, vital signs were: T 36.3°C, P 65 bpm, R 14 bpm, BP 90/60 mmHg. Physical examination showed clear consciousness, moderate pallor, and tenderness in the right lower abdomen without rebound tenderness.

**FIGURE 1:**
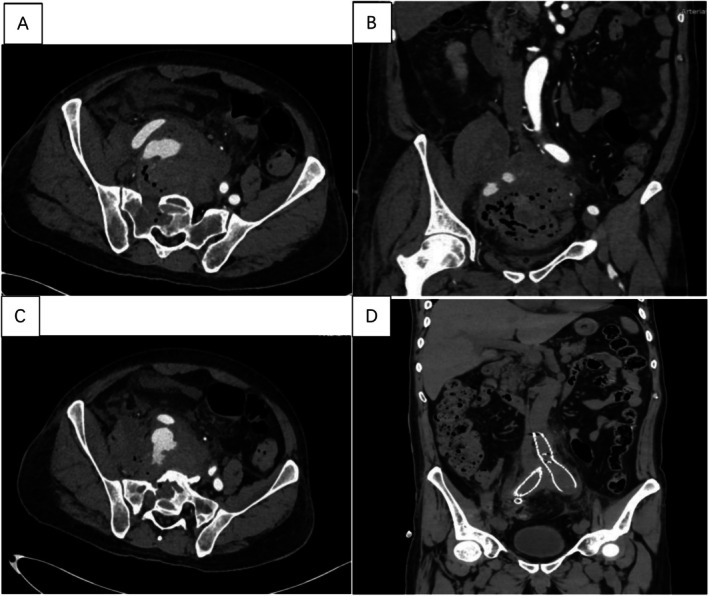
From onset to postoperative abdominal CT imaging findings. (A) Abdominal enhanced CT: Infectious involvement of the sacrum. (B) Abdominal enhanced CT: Abscess formation anterior to the sacrum and within the right lumbar region. (C) Abdominal enhanced CT: Ruptured pseudoaneurysm of the right common iliac artery. (D) Abdominal CT plain scan: Postoperative imaging showing stent graft placement in the infrarenal abdominal aorta and bilateral common iliac arteries.

After admission, the patient developed a high fever (T_max_ 40°C) with chills. Repeat laboratory tests showed: WBC 11.81 × 10^9^/L, neutrophils 91%, hemoglobin 73 g/L, RBC 3.11 × 10^12^/L, platelets 224 × 10^9^/L, elevated CRP, and procalcitonin > 100 ng/mL. Urinalysis revealed RBC 13/μL, WBC 54/μL, occult blood (±), and protein (±). An abdominal enhanced CT demonstrated sacral bone destruction (Figure 1A) with an anterior sacral and right lumbar muscle mass (Figure 1B), highly suggestive of osteomyelitis with invasion into the right common iliac artery, resulting in a ruptured pseudoaneurysm (Figure 1C) approximately 1.5 cm in size.

On Day 1 of admission, following initial management with piperacillin‐tazobactam 4.5 g Q8h and ornidazole 0.5 g Q12h (Days 1–7) for broad‐spectrum antibiotic coverage, along with blood transfusion, shock resuscitation, albumin infusion, and other supportive measures, the patient underwent emergency endovascular repair involving peritoneal stent grafting of the iliac artery aneurysm and embolization of the internal iliac artery. Postoperatively, the patient remained hypotensive and required high‐dose vasopressor support. Bedside ultrasonography revealed significant hemoperitoneum, and diagnostic paracentesis yielded non‐clotting blood. An emergency exploratory laparotomy was performed by the general surgery team. Intraoperative findings included rupture of an internal iliac artery aneurysm with active hemorrhage, intestinal perforation, a recto‐enteric fistula, and a large retroperitoneal abscess. The procedures performed included resection of the internal iliac artery aneurysm, oversewing of the arterial stump, repair of the rectal rupture, creation of a sigmoid loop colostomy, drainage of the retroperitoneal abscess, and placement of abdominal drains. Postoperatively, the patient was maintained on mechanical ventilation and continued on broad‐spectrum antibiotics, vasopressor support, transfusion therapy, and other intensive care measures.

On Day 2, blood cultures returned positive for multidrug‐resistant *E. coli* , while urine cultures were negative. By Day 5, pathological examination of the surgical retroperitoneal specimens revealed the presence of blood clots, hemorrhage, and necrotic tissue.

On Day 8, the patient developed recurrent chills and fever, with a maximum temperature of 39°C. The abdominal drain output became turbid, resembling rice‐water in appearance. The culture of the drainage fluid grew multidrug‐resistant *E. coli* . Antibiotic therapy was escalated to meropenem 1 g Q8h (Days 8–15), after which the patient's mental status improved, body temperature normalized, and oral intake resumed without abdominal pain or discomfort. The abdominal drain output decreased and turned light yellow in color. The enterostomy appeared healthy, with a small amount of brown stool noted.

## 3 Follow‐Up and Outcome

On Day 16, following improvement in infectious parameters, the antimicrobial regimen was de‐escalated to cefoperazone‐sulbactam 3 g Q8h (Days 16–22) in combination with ornidazole 0.5 g Q12h (Days 16–22). A follow‐up abdominal CT showed sacral osteolysis with an anterior sacral and right lumbar muscular mass. Compared with prior imaging from Day 5, the inflammatory involvement had decreased. There was evidence of abdominal and pelvic fluid accumulation, and involvement of the distal right ureter was noted, accompanied by mild hydronephrosis and pelvicalyceal dilation proximally. The findings were advised to be correlated clinically. Additional observations included stents placed in the infrarenal abdominal aorta and bilateral common iliac arteries (Figure 1D), as well as postoperative changes related to the left lower quadrant enterostomy. The patient showed continued clinical improvement and was discharged on Day 22. After discharge, continue to take oral amoxicillin and clavulanate potassium 0.375 g Tid (4 weeks). Due to poor compliance of the patient, the follow‐up visit was not conducted as originally planned. A telephone follow‐up was made, and it was informed that the general condition was good, and there were no more symptoms such as fever, abdominal pain, lumbar pain, or gastrointestinal bleeding. The specific treatment process during hospitalization is shown in Table 1.

**TABLE 1:** The specific treatment process during hospitalization.

Days in hospital	Core event
Day 1	1. Initial treatment: Piperacillin‐tazobactam 4.5 g Q8h and ornidazole 0.5 g Q12h (Days 1–7). 2. Surgical intervention: Emergency endovascular aneurysm repair → open surgical repair.
Day 2	Blood cultures returned positive for multidrug‐resistant *E. coli.*
Day 8	1. Clinical manifestations: Recurrence of high fever and turbidity of abdominal drainage fluid. 2. Laboratory tests: Multi‐drug resistant *E. coli* was cultured in the drainage fluid. 3. Antibiotic adjustment: Upgraded to meropenem 1 g Q8h (Days 8–15).
Day 16	The antibiotic was adjusted to cefoperazone‐sulbactam3g Q8h (Days 16–22) and ornidazole 0.5 g Q12h (Days 16–22).
Day 22	The patient was discharged smoothly and continue to take oral amoxicillin and clavulanate potassium 0.375 g Tid (4 weeks).

## 4 Differential Diagnosis

The patient mainly presented with gastrointestinal bleeding accompanied by septic shock. Laboratory tests indicated significant elevations in inflammatory markers such as WBC, CRP, and PCT. Enhanced abdominal CT revealed an infectious lesion of the bone that invaded the right common iliac artery, resulting in the formation and rupture of a pseudoaneurysm of the right common iliac artery. During emergency laparotomy, rupture bleeding of the iliac artery aneurysm, intestinal perforation, and rectal fistula were found. Blood culture results showed multidrug‐resistant *E. coli* . It was considered that *Escherichia coli* infection led to the formation and rupture of the pseudoaneurysm of the right common iliac artery and concurrent rectal fistula.

At the same time, iatrogenic factors need to be excluded. Some Iatrogenic procedures, such as interventional operations or surgical operations, can also damage the vascular wall and lead to the formation of pseudoaneurysms. This patient had a history of acupuncture, but there were no signs of local skin damage or infection, and no local skin infection was shown on imaging. Therefore, it is not considered that acupuncture caused local infection or bacteremia.

## 5 Discussion

*E. coli* was first isolated and identified by Theodor Escherich in 1885 and subsequently named in his honor [10]. Initially regarded as a commensal member of the gut microbiota, it is now recognized as a major pathogen responsible for a wide range of infections, including those affecting the urinary tract, lungs, bloodstream, wounds, bones, and central nervous system. In China, the intestinal colonization rate of *E. coli* has been rising steadily [11], paralleled by an increasing prevalence of community‐acquired infections caused by extended‐spectrum β‐lactamase (ESBL)‐producing Enterobacteriaceae [12]. The widespread and often inappropriate use of antibiotics has contributed significantly to the emergence of antimicrobial resistance, leading to an increased burden of difficult‐to‐treat and potentially fatal infections globally [13].

The patient's abdominal CT scan revealed destruction of the sacrum and a presacral mass. Further contrast‐enhanced abdominal CT confirmed infectious bone lesions and a psoas abscess. The infection spread, resulting in the formation and rupture of a pseudoaneurysm of the right common iliac artery. Emergency endovascular aneurysm repair (EVAR) was performed. Postoperatively, the patient remained in shock, prompting an exploratory laparotomy. The surgery revealed a large retroperitoneal abscess, a ruptured iliac artery aneurysm, enterobrosis, and a rectal fistula. Surgical exploration not only helped identify the etiology but also allowed direct visualization of the anatomical relationship between the aneurysm rupture and the rectal fistula. Postoperative pathology did not diagnose retroperitoneal fibrosis, tumors, or other diseases. Combined with blood and abdominal drainage fluid culture results, it was indicated that there was multidrug‐resistant *E. coli* . In this case, this patient is more likely to have an *E. coli* ‐infected internal iliac artery aneurysm, which has led to mesenteric ischemia and concurrent rectal fistula. We repeatedly inquired about the medical history of this case, but failed to obtain valuable information. During the hospitalization, the urine culture was also negative, but the fecal culture was not conducted. This is also a regrettable aspect of this case. Rabelo et al. [14] analyzed arterial tissue samples from 36 patients with intracranial aneurysms and found that detection of *E. coli* was associated with aneurysm rupture, further supporting the potential role of *E. coli* in the pathogenesis and rupture of aneurysms.

The patient had a one‐month history of malena. At that time, the medical history was concealed, and the symptoms were atypical. The focus was mainly on low back pain, which was not taken seriously by the patient or the primary hospital, and no endoscopy/colonoscopy was performed. It was not until the onset of bloody stools that the patient suffered from septic shock and rupture of a false aneurysm of the common iliac artery, and surgical intervention was given priority. The management of mycotic aortic aneurysm (MAA) involves prolonged antibiotic therapy combined with surgical intervention [15]. In this case, the patient's vital signs are unstable; EVAR was initially chosen, but the patient still had hemodynamic instability after the operation. Following open surgical repair (OSR) was promptly performed, the removal of the infected lesion was beneficial to the prognosis. The patient's condition stabilized following OSR accompanied by targeted antibiotics, leading to successful discharge. Although EVAR has gained increasing recommendation in recent years, a recent retrospective study [16] indicates that patients with infrarenal infected abdominal aortic aneurysms who undergo prompt OSR benefit from extensive debridement, which facilitates infection control. In cases where OSR is performed after initial EVAR, local encapsulation or even pus invasion around the stent graft is frequently observed. Neither infected tissues nor abscesses can be adequately eradicated through percutaneous drainage or irrigation alone, inevitably elevating the risk of persistent or recurrent infection and contributing to poor long‐term outcomes. Hence, the initial choice of surgical strategy significantly influences prognosis. OSR remains the preferred approach for hemodynamically stable patients with acceptable operative risk. EVAR alone is associated with higher complication rates. For those with ruptured aneurysms, hemodynamic instability, or high surgical risk, EVAR may serve as a bridging measure to be followed by timely definitive OSR.

Studies based on clinical samples in China have demonstrated that multidrug‐resistant *E. coli* exhibits high resistance to trimethoprim‐sulfamethoxazole (SXT), ciprofloxacin (CIP), and ampicillin (AMP) [17], which aligns with the susceptibility profile observed in this patient. Further supporting these findings, a study on antibiotic susceptibility of *E. coli* isolates from neonates in NICUs also revealed high rates of resistance to multiple antibiotics, including cefotaxime (CTX) and trimethoprim‐sulfamethoxazole [18]. Carbapenems remain the last‐line therapy for severe Gram‐negative bacterial infections. The patient was initially treated with piperacillin‐tazobactam and metronidazole for anti‐infection therapy. As the condition worsened and the treatment was adjusted to meropenem, the infection was brought under control, which also confirmed this point.

Through this case, it is evident that in elderly patients with atherosclerosis and a prior history of urinary tract infection, unexplained sepsis should raise clinical suspicion of deep‐seated infection due to *E. coli* . For *E. coli* ‐associated pseudoaneurysms, OSR remains the treatment of choice, complemented by prolonged targeted antibiotic therapy. The optimal duration of antibiotics should be individualized based on clinical follow‐up and radiographic evidence of lesion resolution. Given the growing challenge of antimicrobial resistance, enhanced efforts in monitoring molecular resistance mechanisms of pathogens are imperative to guide effective antimicrobial stewardship.

## Author Contributions

**Xiaojie Zhao:** conceptualization, project administration, resources, writing – original draft. **Ling Zhang:** conceptualization, resources, writing – original draft. **Aiqin Liu:** conceptualization, project administration, writing – review and editing.

## Funding

The authors have nothing to report.

## Consent

A written informed consent was obtained in accordance with the journal's patient consent policy.

## Conflicts of Interest

The authors declare no conflicts of interest.

## Data Availability

The data underpinning the findings of this study are available from the corresponding author upon reasonable request. Public availability of the dataset is limited due to privacy and ethical concerns.
